# Association of sirtuins with clinicopathological parameters and overall survival in gastric cancer

**DOI:** 10.18632/oncotarget.20799

**Published:** 2017-09-08

**Authors:** Xiaobing Shen, Pengfei Li, Yuchao Xu, Xiaowei Chen, Haixiang Sun, Ying Zhao, Mengqi Liu, Wenwen Zhang

**Affiliations:** ^1^ Key Laboratory of Environmental Medicine Engineering, Ministry of Education, School of Public Health, Southeast University, Nanjing, 210009, China

**Keywords:** sirtuins, overall survival, kaplan-meier plotter, gastric cancer

## Abstract

To evaluate the associations of sirtuins (SIRT1-7) with clinicopathological parameters in gastric cancer, sirtuins expression profile in NCBI GEO datasets, GSE62254 and GSE15459, was integrated and analyzed. The results suggested that SIRT4, SIRT6, and SIRT7 were associated with Lauren classification and SIRT3-5 were associated with pStage in gastric cancer. Then an online database derived from 1,065 gastric cancer cases, Kaplan-Meier plotter, was used to explore the associations of the mRNA levels of sirtuins with overall survival in gastric cancer. Survival curves generated from Kaplan-Meier plotter suggested that high expression of SIRT1 mRNA was favorable for overall survival in gastric cancer (SIRT1: HR = 0.64, 95% CI = 0.54–0.76, *P =* 2.2E-07), high expressions of SIRT2-4 and SIRT6-7 were poor for overall survival (SIRT2: HR = 2.31, 95% CI = 1.87–2.87, *P =* 3.6E-15; SIRT3: HR = 1.99, 95% CI = 1.62–2.45, *P =* 2.6E-11; SIRT4: HR = 1.41, 95% CI = 1.19–1.68, *P =* 6.6E-05; SIRT6: HR = 2.02, 95% CI = 1.66–2.47, *P =* 1.7E-12; SIRT7: HR = 1.96, 95% CI = 1.63–2.35, *P =* 2.7E-13), whereas no significant association existed between SIRT5 mRNA expression and overall survival. Further analyses stratified by gender, stages, Lauren classification, differentiation, treatment, and HER2 status were also performed. In summary, high SIRT1 mRNA level was associated with better overall survival, SIRT2-4 and 6–7 were associated with poor overall survival, whereas SIRT5 did not show significant association with overall survival in gastric cancer.

## INTRODUCTION

Gastric cancer remains the major cause of cancer-related death with a bad prognosis [1–3]. Despite recent efforts in multimodal treatment approaches, approximately half of patients diagnosed with advanced gastric cancer still die from recurrent disease after surgical resection or distant metastasis [4, 5]. Now it has been recognized that multiple genetic and epigenetic alterations or abnormality occur in the development of GC [6]. Thus, the identification on the mechanism of initiation, progression, as well as investigation of differential diagnostic prognostic marker and potential drug target, is still needed and will help to provide better prognosis and individualized treatments.

The sirtuins are a family of proteins homologous to yeast silent information regulator 2 (Sir2) and widely expressed in normal tissues in mammary animals. Up to now, seven members have been identified in human (SIRT1–7) and possess nicotinamide adenine dinucleotide (NAD+)-dependent lysine deacetylase (SIRT1, SIRT2, SIRT3, SIRT5, SIRT6, and SIRT7) and mono-ribosyltransferase (SIRT4 and SIRT6) activities [7–14]. Recently, SIRT5 was shown to be a NAD+-dependent protein lysine demalonylase and desuccinylase [15]. The sirtuins play essential roles in cellular physiology including cell metabolism, cell cycle, cell division, and transcriptional regulation and are also involved in the pathogenesis of series of diseases such as metabolic diseases [16], neurodegenerative diseases [17], cardiovascular diseases [18], and aging [19]. Some previous studies have been performed to explore the clinical value and mechanism of sirtuins in gastric cancer. However, the results are inconsistent and not all the seven sirtuins are investigated and compared.

Kaplan-Meier plotter (KM plotter), an online tool, can be utilized to analyze correlations of individual genes with prognosis of patients. This database was initially established using data from a group of 1,809 breast cancer patients [20, 21]. Later, this database also included gene expression data of a total of 1,065 gastric cancer patients derived from NCBI GEO datasets GSE14210, GSE15459, GSE22377, GSE29272, GSE51105, and GSE62254. In this study, we used datasets GSE15459 and GSE62254 to explore the associations of sirtuins with clinicopathological parameters and used KM plotter database to determine the prognostic role of individual sirtuins in human gastric cancer.

## RESULTS

### Correlations of sirtuins with clinicopathological parameters in GC

The associations of sirtuins with the clinicopathological parameters of the patients with GC were firstly explored. We downloaded the datasets, GSE62254 and GSE15459, from NCBI GEO database. Both datasets were consisted of relative large cohort of GC patients and have also been included in the KM plotter database. In GSE62254, SIRT1 and SIRT2 exerted no associations with the clinical features, SIRT3 was associated with T stage, SIRT4 was associated with pStage, SIRT5 was associated with T stage, SIRT6 was associated with age, M stage, and pStage, and SIRT7 was associated with Lauren classification, N stage, and pStage (Table 1). In GSE15459, significant associations were found between Lauren classification and SIRT7, pStage and SIRT2, and pStage and SIRT3 (Table 2). In addition, GSE62254 and GSE15459 were also integrated to investigate the correlations of sirtuins with clinicopathological parameters in GC. The results suggested SIRT4, SIRT6, and SIRT7 were associated with Lauren classification, and SIRT3-5 were associated with pStage (Table 3). Meanwhile, the KM plotter database also included gene expression profile of normal/adjacent tissue from 57 cases. Compare with the normal/adjacent tissues, SIRT2-6 expression in GC cancer tissues was lower while SIRT1 and SIRT7 had no difference (Supplementary Table 1). However, the controls were rare and not each separate dataset was consisted of controls, and the results should further verified.

**Table 1:** Sirtuins and clinicopathological parameters in patients with GC (GSE62254)

Parameters	Cases	SIRT1+	SIRT2+	SIRT3+	SIRT4+	SIRT5+	SIRT6+	SIRT7+
**Gender**								
Male	199	98	96	102	93	98	93	101
Female	101	52	54	48	57	52	57	49
Age								
< 65	161	78	82	78	87	79	70*	81
≥ 65	139	72	68	72	63	71	80	61
**Lauren classification**								
Intestinal	150	75	75	85	69	82	83	85*
Diffuse	142	71	72	62	78	64	62	64
Mixed	8	4	3	3	3	4	5	1
**T stage**								
2	188	88	93	105*	93	104*	100	102
3	91	49	47	34	50	36	38	37
4	21	13	10	11	7	10	12	11
**N stage**								
0	38	20	19	18	16	19	24	10*
1	131	58	60	63	72	71	65	75
2	80	46	43	44	42	42	33	42
3	51	26	28	25	20	18	28	23
**M stage**								
0	273	139	133	138	139	137	143*	133
1	27	11	17	12	11	13	7	17
**pStage**								
1	30	14	14	17	11*	15	22*	8*
2	97	42	46	50	56	55	49	57
3	96	53	49	47	52	49	42	47
4	77	41	41	36	77	31	37	38

*P < 0.05 demonstrated by Chi-square test.

**Table 2:** Sirtuins and clinicopathological parameters in patients with GC (GSE15459)

Parameter	Cases	SIRT1+	SIRT2+	SIRT3+	SIRT4+	SIRT5+	SIRT6+	SIRT7+
**Gender**								
Male	125	63	67	65	66	59	66	63
Female	67	33	29	31	30	37	30	33
**Age**								
< 65	84	42	38	47	40	41	45	42
≥ 65	108	54	58	49	56	55	51	54
**Lauren classification**								
Intestinal	99	43	49	43	50	49	54	60*
Diffuse	75	42	39	45	41	38	33	28
Mixed	18	11	8	8	5	9	9	8
**pStage**								
1	31	17	24*	22*	17	19	15	17
2	29	17	15	15	16	19	15	13
3	72	32	31	39	30	35	39	38
4	60	30	26	20	33	23	27	28

*P < 0.05 demonstrated by Chi-square test.

**Table 3:** Sirtuins and clinicopathological parameters in patients with GC (integrated analysis of GSE62254 and GSE15459)

Parameters	Cases	SIRT1+	SIRT2+	SIRT3+	SIRT4+	SIRT5+	SIRT6+	SIRT7+
**Gender**								
Male	324	161	163	167	159	157	159	164
Female	168	85	83	79	87	89	87	82
**Age**								
< 65	245	120	120	125	127	120	115	123
≥ 65	247	126	126	121	119	126	131	115
**Lauren classification**								
Intestinal	249	118	124	128	119*	131	137*	145*
Diffuse	217	113	111	107	119	102	95	92
Mixed	26	15	11	11	8	13	14	9
**pStage**								
1	61	31	38	39*	28*	34*	37	25
2	126	59	61	65	72	74	64	70
3	168	85	80	86	82	84	81	85
4	137	71	67	56	110	54	64	66

*P < 0.05 demonstrated by Chi-square test.

### Correlations of sirtuins with OS in GC in overall

**Figure 1:**
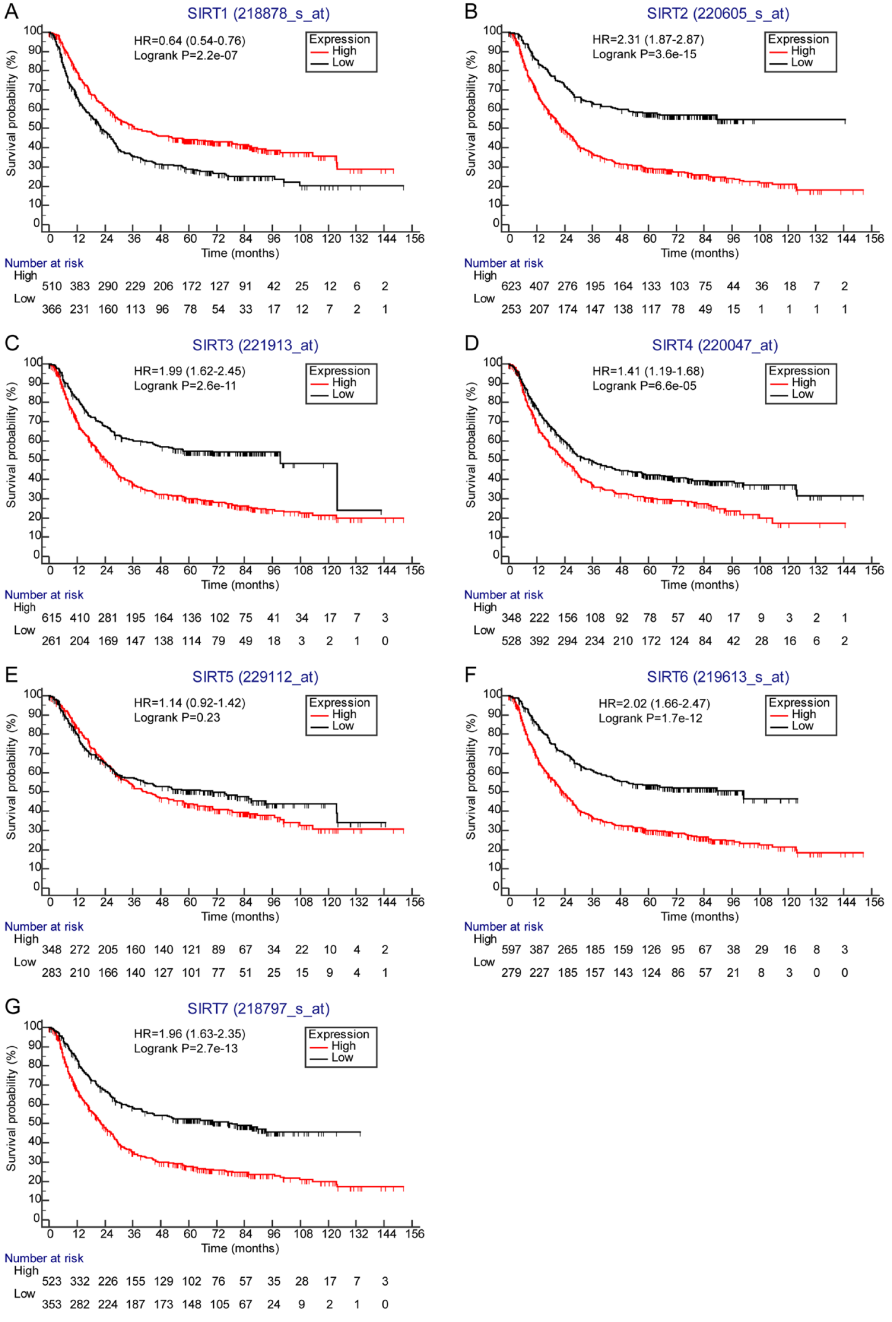
Correlations of sirtuins mRNA expressions with OS in all GC patients (**A**) SIRT1 (*n* = 876). (**B**) SIRT2 (*n* = 876). (**C**) SIRT3 (*n* = 876). (**D**) SIRT4 (*n* = 876). (**E**) SIRT5 (*n* = 631). (**F**) SIRT6 (*n* = 836). (**G**) SIRT7 (*n* = 836).

In overall, 876 GC cases in KM plotter database were available to investigate the correlations of SIRT1-4 and SIRT6-7 with OS while 631 cases were available for SIRT1. Auto select best cutoff value was used to split the patients in survival analyses. Survival curves suggested that high expression of SIRT1 mRNA was favorable for OS (SIRT1: HR = 0.64, 95% CI = 0.54–0.76, *P* = 2.2E-07), high expressions of SIRT2-4 and SIRT6-7 were poor for OS (SIRT2: HR = 2.31, 95% CI = 1.87–2.87, *P* = 3.6E-15; SIRT3: HR = 1.99, 95% CI = 1.62–2.45, *P* = 2.6E-11; SIRT4: HR = 1.41, 95% CI = 1.19–1.68, *P* = 6.6E-05; SIRT6: HR = 2.02, 95% CI = 1.66–2.47, *P* = 1.7E-12; SIRT7: HR = 1.96, 95% CI = 1.63–2.35, *P* = 2.7E-13), and no significant association existed between SIRT5 and OS in GC (Figure 1 and Table 4).

**Table 4:** Correlations of sirtuins with OS in GC in overall

Sirtuins	Cases	HR	95% CI	*P*-value
SIRT1	876	**0.64**	**0.54–0.76**	**2.2E-07**
SIRT2	876	**2.31**	**1.87–2.87**	**3.6E-15**
SIRT3	876	**1.99**	**1.62–2.45**	**2.6E-11**
SIRT4	876	**1.41**	**1.19–1.68**	**6.6E-05**
SIRT5	631	1.14	0.92–1.42	2.3E-01
SIRT6	876	**2.02**	**1.66–2.47**	**1.7E-12**
SIRT7	876	**1.96**	**1.63–2.35**	**2.7E-13**

Then we performed subgroup analyses to explore the effects of clinicopathological features, such as gender, stage, Lauren classification, differentiation, treatment, and HER2 status, on the associations between sirtuins mRNA expression and OS in GC. The effects of gender were firstly investigated. There were 545 male GC patients available for analyzing the correlations between SIRT1-4 and 6-7 and OS while there were 349 male GC patients available for SIRT5. And 236 female patients were available for SIRT1-4 and 6-7 and 187 female patients for SIRT5. Either in male patients or female patients, SIRT1-4 and SIRT6-7 were associated with OS while SIRT5 was not significantly associated with OS (Table 5).

**Table 5:** Correlations of sirtuins with OS in GC stratified by gender

Sirtuins	Gender	Cases	HR	95% CI	*P*-value
SIRT1	Male	545	**0.45**	**0.31–0.66**	**1.7E-05**
	Female	236	**0.63**	**0.51–0.77**	**1.3E-05**
SIRT2	Male	545	**2.08**	**1.38–3.14**	**3.5E-04**
	Female	236	**2.62**	**2.02–3.40**	**6.2E-14**
SIRT3	Male	545	**2.12**	**1.41–3.18**	**2.1E-04**
	Female	236	**2.07**	**1.60–2.67**	**1.8E-08**
SIRT4	Male	545	**1.88**	**1.33–2.68**	**3.3E-04**
	Female	236	**1.46**	**1.16–1.84**	**1.4E-03**
SIRT5	Male	349	0.76	0.48–1.20	2.3E-01
	Female	187	0.80	0.57–1.12	2.0E-01
SIRT6	Male	545	**2.24**	**1.41–3.55**	**4.4E-04**
	Female	236	**2.26**	**1.75–2.92**	**1.6E-10**
SIRT7	Male	545	**1.96**	**1.38–2.79**	**1.2E-04**
	Female	236	**2.12**	**1.68–2.67**	**6.6E-11**

Secondly, subgroup analyses according to stage were performed. In patients with stage 1 cancer, significant association was only found between SIRT2 and OS (*n* = 67, Table 6). In stage 2, SIRT2, 3, 4, 6, and 7 were correlated with OS (*n* = 140). In stage 3, all sirtuins were correlated with OS (*n* = 305 for SIRT1-4 and 6-7, *n* = 197 for SIRT5). And in stage 4, significant association was identified between SIRT2, 5, 6, and 7 and OS (*n* = 140). Notably, unlike the results in overall, SIRT5 was significantly associated with OS in stage 3 and stage 4, however, exerting opposite effects on OS (stage 3, n=197, HR=1.70, 95% CI = 1.16–2.49, *P* = 6.3E-03; stage 4, *n* = 140, HR = 0.63, 95% CI = 0.42–0.94, *P* = 2.2E-02).

**Table 6:** Correlations of sirtuins with OS in GC stratified by stages

Sirtuins	Stage	Cases	HR	95% CI	*P*-value
SIRT1	1	67	0.38	0.14–1.06	5.6E-02
	2	140	1.55	0.78–3.06	2.1E-01
	3	305	**0.59**	**0.43–0.81**	**9.4E-04**
	4	148	0.81	0.55–1.19	2.8E-01
SIRT2	1	67	**4.71**	**1.33–16.74**	**8.5E-03**
	2	140	**2.55**	**1.37–4.76**	**2.3E-03**
	3	305	**2.60**	**1.79–3.77**	**2.1E-07**
	4	148	**1.65**	**1.11–2.46**	**1.3E-02**
SIRT3	1	67	**11.02**	**1.44–84.40**	**3.9E-03**
	2	140	**3.36**	**1.69–6.70**	**2.5E-04**
	3	305	**2.21**	**1.52–3.21**	**1.8E-05**
	4	148	0.76	0.50–1.14	1.8E-01
SIRT4	1	67	1.88	0.70–5.08	2.1E-01
	2	140	**2.25**	**1.22–4.18**	**8.1E-03**
	3	305	**1.43**	**1.08–1.90**	**1.3E-02**
	4	148	1.32	0.89–1.97	1.6E-01
SIRT5	1	62	3.00	0.66–13.58	1.3E-01
	2	135	1.91	0.91–4.03	8.1E-02
	3	197	**1.70**	**1.16–2.49**	**6.3E-03**
	4	140	**0.63**	**0.42–0.94**	**2.1E-02**
SIRT6	1	67	2.63	0.083–8.29	8.7E-02
	2	140	**2.69**	**1.42–5.12**	**1.7E-03**
	3	305	**1.90**	**1.42–2.54**	**1.0E-05**
	4	148	**1.49**	**1.02–2.2**	**3.9E-02**
SIRT7	1	67	2.76	0.94–8.08	5.4E-02
	2	140	**2.03**	**1.11–3.72**	**1.9E-02**
	3	305	**1.81**	**1.33–2.46**	**1.3E-04**
	4	148	**1.60**	**1.05–2.45**	**2.8E-02**

Thirdly, Lauren classification was used to stratify the patients. SIRT1, 2, 3, 6, and 7 were found to be associated with OS in intestinal GC (*n* = 320, Table 7). SIRT2, 3, and 4 were associated with OS in diffuse GC (*n* = 141). And, only SIRT3 was associated with OS in mixed GC (*n* = 32).

**Table 7:** Correlations of sirtuins with OS in GC stratified by lauren classification

Sirtuins	Lauren classification	Cases	HR	95% CI	*P*-value
SIRT1	Intestinal	320	**0.51**	**0.37–0.70**	**2.1E-05**
	Diffuse	141	1.26	0.88–1.81	2.1E-01
	Mixed	32	0.43	0.15–1.22	1.0E-01
SIRT2	Intestinal	320	**3.39**	**2.32–4.95**	**2.4E-11**
	Diffuse	141	**1.71**	**1.17–2.51**	**5.4E-03**
	Mixed	32	2.02	0.57–7.23	2.7E-01
SIRT3	Intestinal	320	**2.74**	**1.89–3.96**	**2.4E-08**
	Diffuse	141	**1.44**	**1.01–2.04**	**4.3E-02**
	Mixed	32	**4.07**	**1.14–14.47**	**1.9E-02**
SIRT4	Intestinal	320	**1.37**	**1.00–1.88**	**4.8E-02**
	Diffuse	141	**1.59**	**1.13–2.24**	**7.1E-03**
	Mixed	32	2.42	0.86–6.85	8.6E-02
SIRT5	Intestinal	169	1.32	0.90–1.93	1.5E-01
	Diffuse	140	0.79	0.56–1.12	1.8E-01
	Mixed	29	0.37	0.11–1.20	8.4E-02
SIRT6	Intestinal	320	**3.03**	**2.05–4.48**	**4.9E-09**
	Diffuse	141	1.38	0.98–1.95	6.8E-02
	Mixed	32	2.31	0.77–6.94	1.2E-01
SIRT7	Intestinal	320	**2.71**	**1.91–3.83**	**5.1E-09**
	Diffuse	141	1.37	0.97–1.92	7.1E-02
	Mixed	32	2.40	0.83–6.95	9.7E-02

Furthermore, subgroup analyses according to differentiation or treatment were also performed, respectively. In GC with poor differentiation, significant associations were identified between SIRT4-5 and OS (*n* = 165 for SIRT4; *n* = 121 for SIRT5, Table 8). And significant association was only found between SIRT3 and OS in GC with moderated differentiation (*n* = 67). In GC with well differentiation, SIRT3 and 7 was associated with OS (*n* = 32). For association between SIRT5 and OS in GC with well differentiation, analysis could not be conducted due to the case number was very small (*n* = 5). After grouping by treatment (surgery alone, 5-FU adjuvant therapy, or other adjuvant therapy), significant associations were found between SIRT2-4 and OS in GC patients underwent surgery alone (*n* = 380, Table 9). In patients underwent 5-FU adjuvant therapy, significant associations were found between SIRT1, 2, 5, and 7 and OS (*n* = 153, Table 9). And significant associations were found between SIRT6 and OS in patients underwent other adjuvant therapy (*n* = 76, Table 9).

**Table 8:** Correlations of sirtuins with OS in GC stratified by differentiation

Sirtuins	Differentiation	Cases	HR	95% CI	P-value
SIRT1	Poor	165	0.71	0.46–1.10	1.2E-01
	Moderate	67	0.62	0.32–1.21	1.6E-01
	Well	32	0.50	0.19–1.30	1.5E-01
SIRT2	Poor	165	0.72	0.48–1.10	1.3E-01
	Moderate	67	1.43	0.72–2.83	3.1E-01
	Well	32	0.44	0.15–1.31	1.3E-01
SIRT3	Poor	165	1.24	0.83–1.85	2.9E-01
	Moderate	67	**0.47**	**0.25–0.90**	**2.0E-02**
	Well	32	**3.89**	**1.59–9.49**	**1.5E-03**
SIRT4	Poor	165	**1.79**	**1.19–2.68**	**4.2E-03**
	Moderate	67	0.56	0.27–1.14	1.0E-01
	Well	32	0.45	0.18–1.15	8.8E-02
SIRT5	Poor	121	**0.57**	**0.35–0.93**	**2.1E-02**
	Moderate	67	1.42	0.72–2.81	3.1E-01
	Well	5	NA		
SIRT6	Poor	165	0.75	0.49–1.12	1.6E-01
	Moderate	67	1.75	0.9–3.42	9.5E-02
	Well	32	0.62	0.25–1.55	3.1E-01
SIRT7	Poor	165	1.37	0.88–2.12	1.6E-01
	Moderate	67	1.61	0.67–3.88	2.8E-01
	Well	32	**0.30**	**0.10–0.90**	**2.3E-02**

**Table 9:** Correlations of sirtuins with OS in GC stratified by treatment

Sirtuins	Treatment	Cases	HR	95% CI	*P*-value
SIRT1	Surgery alone	380	1.20	0.89–1.62	2.4E-01
	5-FU adjuvant	153	**0.64**	**0.44–0.95**	**2.4E-02**
	Other adjuvant	76	1.99	0.72–5.47	1.8E-01
SIRT2	Surgery alone	380	**1.55**	**1.15–2.08**	**3.7E-03**
	5-FU adjuvant	153	**0.68**	**0.48–0.96**	**2.8E-02**
	Other adjuvant	76	6.10	1.41–26.33	5.7E-03
SIRT3	Surgery alone	380	**1.47**	**1.09–1.97**	**1.1E-02**
	5-FU adjuvant	153	0.71	0.48–1.05	8.3E-02
	Other adjuvant	76	1.66	0.68–4.05	2.6E-01
SIRT4	Surgery alone	380	**1.37**	**1.02–1.82**	**3.3E-02**
	5-FU adjuvant	153	1.25	0.85–1.84	2.5E-01
	Other adjuvant	76	0.60	0.22–1.66	3.2E-01
SIRT5	Surgery alone	380	1.26	0.94–1.69	1.2E-01
	5-FU adjuvant	34	0.23	0.08–0.68	3.7E-03
	Other adjuvant	76	0.75	0.31–1.80	5.1E-01
SIRT6	Surgery alone	380	1.33	1.00–1.79	5.2E-02
	5-FU adjuvant	153	1.26	0.89–1.79	1.9E-01
	Other adjuvant	76	**0.36**	**0.15–0.88**	**2.0E-02**
SIRT7	Surgery alone	380	**1.35**	**1.01–1.80**	**4.3E-02**
	5-FU adjuvant	153	**1.60**	**1.12–2.27**	**8.4E-03**
	Other adjuvant	76	0.59	0.24–1.43	2.4E-01

Finally, effects of HER2 status on the associations between sirtuins and OS in GC were evaluated. In GC with negative HER2 status, all sirtuins except SIRT5 and SIRT7 were associated with OS (*n* = 532, Table 10) and in GC with positive HER2 status, all sirtuins except SIRT4 were associated with OS (*n* = 344 for SIRT1-4 and 6-7; *n* = 202 for SIRT5).

**Table 10:** Correlations of sirtuins with OS in GC stratified by HER2 status

Sirtuins	HER2 status	Cases	HR	95% CI	*P*-value
SIRT1	Negative	532	**0.64**	**0.51–0.81**	**1.4E-04**
	Positive	344	**0.58**	**0.42–0.80**	**7.3E-04**
SIRT2	Negative	532	**2.20**	**1.71–2.83**	**3.9E-10**
	Positive	344	**1.58**	**1.14–2.18**	**5.5E-03**
SIRT3	Negative	532	**1.68**	**1.31–2.15**	**3.2E-05**
	Positive	344	**1.52**	**1.11–2.06**	**7.7E-03**
SIRT4	Negative	532	**1.60**	**1.27–2.00**	**4.7E-05**
	Positive	344	1.28	0.98–1.67	7.0E-02
SIRT5	Negative	429	0.83	0.62–1.12	2.2E-01
	Positive	202	0.63	**0.43–0.94**	**2.2E-02**
SIRT6	Negative	532	1.81	**1.43–2.29**	**5.2E-07**
	Positive	344	1.76	**1.28–2.41**	**3.8E-04**
SIRT7	Negative	532	1.91	**1.53–2.40**	**1.0E-08**
	Positive	344	1.60	**1.23–2.07**	**3.8E-04**

In addition, the online database also included the first progression (FP) information of the GC patients, and the analyses results suggested that high expression of SIRT1 was associated with long FP time while high expression of SIRT2-4 and SIRT6-7 was associated with short FP time and no significant association was found between SIRT5 and FP in GC in overall (Supplementary Figure 1 and Supplementary Table 2).

## DISCUSSION

Gastric cancer (GC) is main cause of cancer related death and presents high mortality rate among all digestive tract malignancies due to chemoradiotherapy resistance and distant metastasis. Thus it is crucial to reveal pathogenesis of GC and find novel prognostic strategies, early diagnostic tools, and effective therapeutic approaches. Here, we used an online database to explore the clinical value of sirtuins in predicting overall survival (OS) in GC.

Of all the seven sirtuins, SIRT1 was the most studied one in GC. A series of studies have investigated the associations of SIRT1 with OS in GC. Of which, four studies reported that high SIRT1 expression was associated with poor OS [22–25] while one study reported that there was a trend of association between SIRT1 with good OS [26]. A meta-analysis combing the previous studies suggested that high SIRT1 expression was closely linked with the 3-year OS (OR = 0.25, 95% CI = 0.16–0.39, *P* < 0.00001, fixed model) while it was not associated with 5-year OS (OR = 0.44, 95% CI = 0.15–1.28, *P* = 0.13, random model) [27]. In our study, we found that high expression of SIRT1 mRNA was favorable for OS (SIRT1: HR = 0.64, 95% CI = 0.54–0.76, *P* = 2.2E-07). The inconsistency of these results might be due to the heterogeneity of these studies such as sample size, cancer site, and cutoff value. Several meta-analyses have also been performed to evaluate the associations of SIRT1 with OS in other solid carcinomas. All the meta-analyses studies revealed a significant association between high SIRT1 with poor OS in these solid carcinomas including breast cancer [28], hepatocellular carcinoma [29], colorectal cancer [30], lung cancer [31, 32], liver cancer [32]. Wang et al. also have pooled all the eligible data of solid malignancies and identified the same conclusion [32]. Mechanism researches suggested that SIRT1 can counteract the activation of STAT3 and NF-κB to repress cell growth [33] and lead to G1-phase arrest via NF-κB/Cyclin D1 signaling in gastric cancer[34]. The previous studies suggested that SIRT1 was also the target of certain microRNAs such as miR-543 and miR-204. miR-543 can promote gastric cancer cell proliferation by targeting SIRT1[35] and miR-204 can down-regulate SIRT1 and revert SIRT1-induced epithelial-mesenchymal transition, anoikis resistance, and invasion in gastric cancer[36].

For SIRT2, a previous study indicated that combination of SIRT2 with other three genes could be used to predict OS in GC[37]. Here, we identified that high expression of SIRT2 expression was a biomarker of worse OS in GC patients (HR = 2.31, 95% CI = 1.87–2.87, *P* = 3.6E-15).

Up to now, there were three studies reporting the association of SIRT3 with OS in GC. The association was significant in two studies [38, 39] and insignificant in one study [40]. A meta-analysis has pooled the previous two studies and found increased SIRT3 expression was associated with better OS in GC, however, the total included cases were only 286 [41]. Our results suggested that high SIRT3 mRNA expression was associated with poor prognosis in GC (HR = 1.99, 95% CI = 1.62–2.45, *P* = 2.6E-11). The inconsistency needed to be verified in further studies with large scale of patients. Function studies revealed that SIRT3 plays dual role in GC development. Wang et al. reported that SIRT3 can inhibit cell proliferation in human gastric cancer through down-regulation of Notch-1 [42] whereas Cui et al. reported that SIRT3 can enhance glycolysis and proliferation in SIRT3-expressing GC Cells [43].

The study performed by Huang et al. suggested SIRT4 is associated with some clinicopathological features in GC but did not reported the association of SIRT4 with prognosis [44]. In the present study, we found that high SIRT4 mRNA expression exhibited a significant associations with Lauren classification, pStage, and OS in GC (OS: HR = 1.41, 95% CI = 1.19–1.68, *P* = 6.6E-05).

Currently, SIRT5 and 6 have not been investigated in gastric cancer, but have been shown to be involved in other cancers, such as breast cancer, lung cancer, hepatocellular carcinoma, head and neck squamous cell carcinoma, and ovarian cancer [45–50]. Our study revealed that high SIRT6 expression was associated with poor OS in GC (HR = 2.02, 95% CI = 1.66–2.47, *P* = 1.7E-12) while SIRT5 was not correlated with OS (HR=1.14, 95% CI=0.92–1.42, *P* = 0.23). Interestingly, after stratification by stage or differentiation, high SIRT5 mRNA expression was associated with OS in stage 3 (HR=1.70, 95% CI = 1.16–2.49, *P* = 6.3E-03), stage 4 (HR = 0.63, 95% CI = 0.42–0.94), and poor differentiated GC (HR = 0.57, 95% CI = 0.35–0.93, *P* = 2.1E-02). The different effects of SIRT5 on OS in GC with different stages or differentiations should be checked in more large cohorts.

Involvement of SIRT7 in GC was reported in only one study that it can promote gastric cancer growth and inhibit apoptosis by epigenetically inhibiting miR-34a and is associated with poor prognosis [51]. Here, we also identified SIRT7 as a biomarker for poor OS in GC (HR = 1.73, 95% CI = 1.45–2.05, *P* = 3.2E-10).

## MATERIALS AND METHODS

To investigate the associations of sirtuins mRNA levels with clinicopathological parameters, we downloaded gastric cancer mRNA profile data and corresponding clinical data from publicly available GEO database. GSE62254 and GSE15459 with large number of gastric cancer patient samples and complete clinical information were selected, which had been also included in the Kaplan-Meier plotter database. The clinical samples were stratified into two groups by sirtuins mRNA levels with the median as cut-off value. The Chi-square test or Fisher’s exact test were performed to explore the correlations between sirtuins expression and clinicopathological parameters. Statistical analyses were conducted with the software GraphPad Prism 6. *P* value less than 0.05 was considered statistically significant.

Then we used the Kaplan-Meier plotter (www.kmplot.com) to investigate the predictive value of mRNA expressions of sirtuins in overall survival in gastric cancer. Currently, the Kaplan-Meier plotter is capable to assess the effect of 54, 675 genes on survival of 10, 188 clinical cancer samples, including 4, 142 breast, 1, 648 ovarian, 2, 437 lung and 1, 065 gastric cancer patients[52]. Briefly, the seven sirtuins (SIRT1, SIRT2, SIRT3, SIRT4, SIRT5, SIRT6, and SIRT7) were entered into the database (http://kmplot.com/analysis/index. php?p = service and cancer = gastric) to obtain Kaplan-Meier plots in which the number-at-risk is indicated below the main plot. The Affy IDs of SIRT1-7 were 218878_s_at (SIRT1), 220605_s_at (SIRT2), 221913_at (SIRT3), 220047_at (SIRT4), 229112_at (SIRT5), 219613_s_at (SIRT6), and 218797_s_at (SIRT7), respectively. If the gene had multiple chip probe sets, JetSet best probe set was selected. The patient samples were divided into two groups according to the mRNA expression with auto select best cutoff value (high vs. low expression). The hazard ratio (HR) with 95% confidence intervals and log rank *p* value was calculated and displayed on the webpage. HER2 status was determined using the gene chip probe set 216836_s_at as described before [21].

## CONCLUSIONS

To investigate and compare the clinical value of sirtuins in predicting overall survival in GC, we analyze the associations of all sirtuins (SIRT1-7) mRNA expressions with overall survival in GC using an online database, KM plotter. And the results suggested that high SIRT1 mRNA level was associated with better overall survival, SIRT2-4 and 6-7 were associated with poor overall survival, whereas SIRT5 did not show significant association with overall survival in GC.

## SUPPLEMENTARY MATERIALS FIGURE AND TABLES


