# Supplementary material for: Association of sirtuins with clinicopathological parameters and overall survival in gastric cancer
Source: Oncotarget. 2017 Sep 8;8(43):74359–70. doi: 10.18632/oncotarget.20799 (PMC5650347; doi:10.18632/oncotarget.20799)
Supplement: Supplementary file 1 [file oncotarget-08-74359-s001.pdf]

Association of sirtuins with clinicopathological parameters and overall survival in gastric cancer

SUPPLEMENTARY MATERIALS

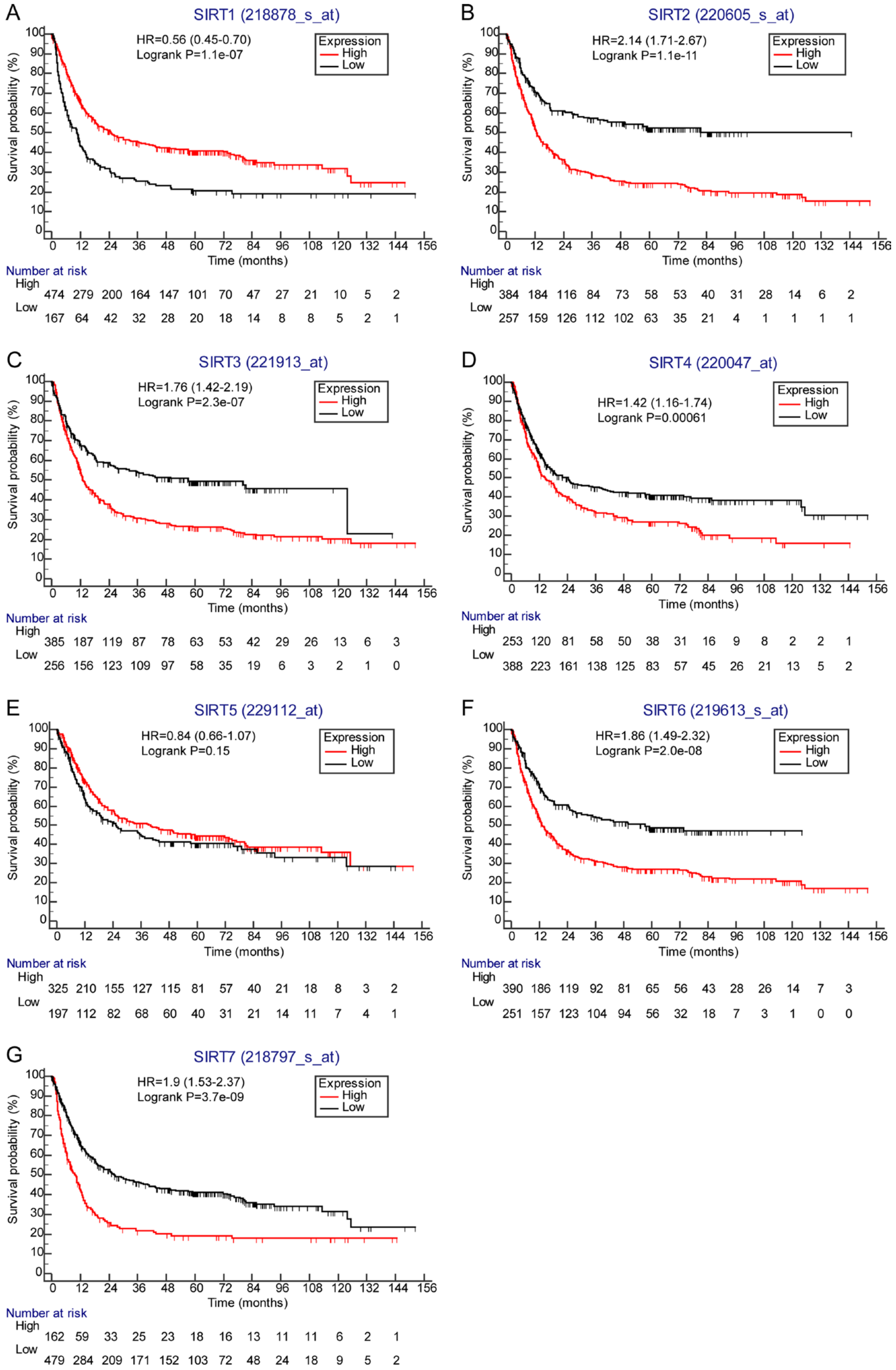

Supplementary Figure 1: Correlations of sirtuins mRNA expressions with first progression time in all GC patients. (**A**) SIRT1 (n = 641). (**B**) SIRT2 (n = 641). (**C**) SIRT3 (n = 641). (**D**) SIRT4 (n = 641). (**E**) SIRT5 (n = 522). (**F**) SIRT6 (n = 641). (**G**) SIRT7 (n = 641).

Supplementary Table 1: Comparison of sirtuins expression in GC tissues with normal/adjacent tissues

Sirtuins	Cancer/Normal	Min	Q1	Median	Q3	Max	*P* value
SIRT1	Normal	332	622	749	978	1182	2.54E-01
	Cancer	73	533	716	922	3816	
SIRT2	Normal	235	618	828	1127	2241	**1.25E-07**
	Cancer	72	313	502	829	2534	
SIRT3	Normal	19	92	171	209	571	**4.95E-02**
	Cancer	4	66	118	245	1077	
SIRT4	Normal	5	27	75	104	235	**1.56E-04**
	Cancer	1	19	39	62	313	
SIRT5	Normal	28	56	83	131	287	**7.24E-03**
	Cancer	4	50	72	95	199	
SIRT6	Normal	93	207	311	389	856	**1.29E-03**
	Cancer	22	135	211	359	1246	
SIRT7	Normal	281	551	794	1002	1497	4.77E-01
	Cancer	177	478	723	994	3245	

Supplementary Table 2: Correlations of sirtuins with first progression in GC in overall

Sirtuins	Cases	HR	95% CI	*P*-value
SIRT1	641	**0.56**	**0.45–0.70**	**1.1E-07**
SIRT2	641	**2.14**	**1.71–2.67**	**1.1E-11**
SIRT3	641	**1.76**	**1.42–2.19**	**2.3E-07**
SIRT4	641	**1.42**	**1.16–1.74**	**6.1E-05**
SIRT5	522	0.84	0.66–1.07	1.5E-01
SIRT6	641	**1.86**	**1.49–2.32**	**2.0E-08**
SIRT7	641	**1.9**	**1.53–2.37**	**3.7E-09**
